# Gut microbial and functional signatures in breast cancer: an integrated metagenomic and machine learning approach to non-invasive detection

**DOI:** 10.3389/fmicb.2025.1722632

**Published:** 2026-01-15

**Authors:** Yalin Li, Yi Cheng, Weichi Liu, Jingjin Li, Shiqi Li, Suriguga, Teng Ma, Lai-Yu Kwok, Zhihui Cai, Zhihong Sun

**Affiliations:** 1 Inner Mongolia Key Laboratory of Dairy Biotechnology and Engineering, Inner Mongolia Agricultural University, Hohhot, Inner Mongolia, China; 2 Key Laboratory of Dairy Products Processing, Ministry of Agriculture and Rural Affairs, Inner Mongolia Agricultural University, Hohhot, Inner Mongolia, China; 3 Key Laboratory of Dairy Biotechnology and Engineering, Ministry of Education, Inner Mongolia Agricultural University, Hohhot, Inner Mongolia, China; 4 Graduate School of Baotou Medical College, Inner Mongolia University of Science and Technology, Baotou, Inner Mongolia, China; 5 Department of Oncology, Inner Mongolia People's Hospital, Hohhot, Inner Mongolia, China

**Keywords:** case-control study, diagnostic biomarker, dysbiosis, gut virome, shotgun metagenomics, microbiome-host interactions, predictive modeling

## Abstract

**Introduction:**

Breast cancer is associated with significant restructuring of the gut ecosystem. Gut microbial composition and function may influence cancer development and progression through immune modulation, metabolic regulation, and inflammation-related pathways.

**Methods:**

Using shotgun metagenomic sequencing of fecal samples from 38 stage I–III breast cancer patients and 36 age- and body mass index-matched healthy controls. Machine learning models were constructed to evaluate the diagnostic potential of integrated microbial and metabolic features.

**Results:**

Significant alterations were observed in gut microbiota composition, including depletion of beneficial taxa (*Limosilactobacillus fermentum, Blautia* sp.) and enrichment of *Prevotella copri*. Pathways involved in short-chain fatty acid and purine metabolism were reduced. The gut phageome exhibited structural changes and altered correlations with bacterial hosts. Predictive analysis revealed depletion of short-chain fatty acids (butyrate, propionate), purine intermediates (hypoxanthine, xanthine), and nicotinate in patients. A machine learning model integrating microbial and predicted metabolic features achieved an area under the curve values of 0.78 in the discovery cohort and 0.73 (recall = 0.74) in an independent validation cohort.

**Discussion:**

Coordinated gut microbiome, phageome, and metabolome alterations characterize breast cancer, offering potential non-invasive biomarkers and mechanistic insights for disease detection and intervention.

## 1 Introduction

Breast cancer (BC) is the most commonly diagnosed malignancy among women worldwide, accounting for approximately 25% of all female cancer cases. In 2022 alone, an estimated 2.3 million new cases were reported, representing 11.6% of all cancer diagnoses globally (Bray et al., 2024). Despite advances in early detection and treatment, both the incidence and mortality rates of BC continue to rise, underscoring the urgent need to better understand its underlying etiology and identify novel risk factors and therapeutic targets. Established risk factors for BC include age, genetics, hormonal and reproductive factors, lifestyle choices, and psychosocial stress. However, recent research has highlighted the potential role of the human microbiome as a previously underappreciated contributor to cancer susceptibility and progression (Kim et al., 2025; Sun et al., 2017).

The human body hosts a vast and dynamic community of microorganisms, collectively known as the microbiota, with the gastrointestinal tract serving as the largest reservoir. The gut microbiome, the complete genetic repertoire of these microbial communities, forms a complex and functionally rich ecosystem often referred to as the “second genome” (Zhu et al., 2010). Bacteria dominate this ecosystem, constituting over 99% of the gut microbial population. These microbes engage in bidirectional communication with the host through neural, endocrine, immune, and metabolic pathways, playing critical roles in maintaining homeostasis. For instance, intestinal plasma cells interact directly with gut microbes to regulate microbial colonization, while commensal bacteria inhibit pathogen invasion through competitive exclusion and suppression of epithelial adhesion, thereby reinforcing mucosal defense (Woelfel et al., 2024; Yu et al., 2012). Under healthy conditions, a dynamic equilibrium exists between the host and its microbiota, fostering mutualism and supporting essential physiological functions. Disruption of this balance, termed dysbiosis (Fassarella et al., 2021), can lead to chronic inflammation, immune dysregulation, genomic instability, and ultimately, disease pathogenesis, including carcinogenesis.

Emerging evidence underscores the involvement of the gut microbiome in BC development and progression. Patients with BC exhibit distinct alterations in gut microbial composition, characterized by increased abundance of *Clostridiaceae, Faecalibacterium*, and *Ruminococcaceae*, and reduced levels of *Dorea* and *Lachnospiraceae* (Goedert et al., 2018). Notably, the presence of toxin-producing *Bacteroides fragilis* in either the gut or breast tissue has been linked to enhanced tumor invasiveness, promotion of cancer cell self-renewal, and facilitation of distant metastasis. Importantly, gut dysbiosis may not only be a consequence of cancer but also a modulator of therapeutic response (Ma et al., 2024). For instance, in epithelial ovarian cancer, platinum-responsive patients display a more diverse and stable gut microbiome enriched with *Veillonellaceae*, a family of lactate-utilizing bacteria (D'Amico et al., 2021). Furthermore, specific microbial taxa have shown promise as prognostic biomarkers: *Methanobrevibacter smithii*, members of *Eubacteriaceae*, and *Akkermansia* are more prevalent in post-chemotherapy patients with early-stage tumors (such as N0 or T1), suggesting a protective or modulatory role in disease outcomes (Terrisse et al., 2021). Notably, the gut microbiome may influence breast cancer risk in part through its regulatory effects on host estrogen metabolism, referred to as the estrobolome. The estrobolome encompasses the collection of gut microbial genes involved in estrogen metabolism that can modulate systemic estrogen levels. Specifically, estrogens are conjugated in the liver to inactive glucuronide conjugates and excreted into the intestine via bile. Certain intestinal bacteria expressing β-glucuronidase can deconjugate these metabolites, releasing active estrogens that are subsequently reabsorbed through the intestinal mucosa (Funk et al., 2023). This enterohepatic recycling increases systemic estrogen exposure and may elevate breast cancer risk. Bacteria capable of producing β-glucuronidase include members of the genera *Akkermansia, Bacteroides, Bifidobacterium, Collinsella*, and *Lactobacillus* (Edwinson et al., 2022). Consequently, alterations in gut microbial composition may influence estrogen metabolic pathways and play a pivotal role in the development and progression of hormone receptor–positive breast cancer.

Beyond microbial composition, microbial metabolites are increasingly recognized as key mediators in BC pathophysiology. Indolepropionic acid, a tryptophan-derived metabolite produced by gut bacteria, is significantly reduced in BC patients. Experimental studies have demonstrated that indolepropionic acid supplementation inhibits tumor cell proliferation in murine models, increases oxidative stress in cancer cells, and suppresses the population of cancer stem cells (Sári et al., 2020). Conversely, elevated levels of *Clostridium* species and their metabolite trimethylamine N-oxide have been detected in immunologically active breast tumors. Trimethylamine N-oxide promotes ferroptosis in tumor cells via activation of the protein kinase R-like endoplasmic reticulum kinase (PERK), thereby enhancing CD8^+^ T cell-mediated antitumor immunity *in vivo* (Wang et al., 2022). Moreover, gut microbiota influence T cell differentiation, with a progressive increase in regulatory T cells observed during BC progression. The abundance of Foxp3^+^ regulatory T cells has been strongly associated with both recurrence-free and overall survival, underscoring their potential prognostic value (Crusz and Balkwill, 2015).

Recent advances in high-throughput sequencing, particularly shotgun metagenomics, have revolutionized the study of the gut microbiome by enabling culture-independent, genome-resolved characterization of microbial communities. These technologies provide comprehensive insights into both taxonomic composition and functional potential, facilitating the identification of disease-associated microbial signatures with high precision (Jin et al., 2025). Building on this progress, we conducted a case-control study involving 38 patients with stage I–III breast cancer and 36 age- and body mass index-matched healthy controls (HCs) as a discovery cohort. Shotgun metagenomic sequencing was employed to characterize gut microbiome alterations associated with BC and to identify potential microbial and metabolic biomarkers. Our primary objectives were to: (1) delineate disease-specific gut microbial and metabolic signatures; and (2) develop and validate a microbiome-based diagnostic model using machine learning in an independent external cohort. These findings are expected to provide a theoretical foundation for the development of non-invasive, microbiome-targeted strategies in BC screening, prognosis, and therapeutic monitoring.

## 2 Materials and methods

### 2.1 Participant recruitment

Participants were recruited from the Oncology Center of Inner Mongolia People's Hospital between June 2023 and January 2024. Female patients diagnosed with stage I–III BC within the preceding month were considered for inclusion. Written informed consent was obtained from all individuals who met the preliminary eligibility criteria.

The inclusion criteria were defined as follows: (1) females aged 18–65 years with a pathological diagnosis of BC, scheduled to undergo modified radical mastectomy and to receive an AC (Adriamycin and cyclophosphamide)-based chemotherapy regimen; (2) absence of organic psychiatric disorders and no brain metastasis; (3) an Eastern Cooperative Oncology Group (ECOG) performance status score of at least 0 or 1, with a life expectancy exceeding 6 months; and (4) no use of antidepressants or antipsychotic drugs within the 2 weeks prior to enrollment.

Exclusion criteria included: (1) concurrent radiotherapy; (2) confirmed radiological disease progression necessitating treatment discontinuation; (3) pregnancy during the study period; (4) presence of significant clinical adverse events, laboratory abnormalities, or comorbidities that could preclude further treatment or diminish clinical benefit; (5) substantial deterioration in general health that would impair trial participation; (6) antibiotics, probiotics, and prebiotics were used for at least 2 weeks prior to enrollment; and (7) inability to read or provide written informed consent.

A total of 38 patients fulfilled the eligibility criteria and were enrolled. During the same period, 36 healthy female controls, matched for age and body mass index, were recruited from the Health Check-up Center of Inner Mongolia People's Hospital (Table 1).

**Table 1:** Baseline demographic and clinical characteristics of breast cancer patients and healthy controls.

**Characteristic**	**Breast cancer patients (BCs; *N* = 38)**	**Healthy controls (HCs; *N* = 36)**	***P*-value**
Age, years (SD)	51.15 (8.78)	49.29 (9.87)	0.85
Body mass index, kg/m^2^ (SD)	22.83 (2.48)	22.79 (1.97)	0.71
**Educational level**, ***n*** **(%)**			
Junior high school or below	9 (23.08%)	10 (28.57%)	0.82
Senior high school	20 (51.28%)	18 (51.43%)	0.75
College or higher	10 (25.62%)	7 (20%)	0.47
**Menopausal status**, ***n*** **(%)**			
Postmenopausal	21 (55.26%)	19 (52.78%)	0.75
Premenopausal	17 (44.74%)	17 (47.22%)	1
**Tumor stage**, ***n*** **(%)**			
I	16 (42.11%)	N/A	
II	14 (36.84%)	N/A	
III	8 (21.05%)	N/A	
**Molecular subtype**, ***n*** **(%)**			
Luminal A	4 (10.53%)	N/A	
Luminal B	22 (57.89%)	N/A	
HER2-enriched	6 (15.79%)	N/A	
Tri-negative	6 (15.79%)	N/A	
Smoking, *n* (%)	3 (7.89%)	4 (11.11%)	0.7
Alcohol consumption, *n* (%)	1 (2.63)	0	0.3

Age and BMI were compared using the Wilcoxon rank-sum test; categorical variables (e.g., menopausal status, education, smoking, alcohol use) were analyzed using the chi-square test.

N/A, not applicable.

### 2.2 Fecal sample collection and metagenomic sequencing

Fecal samples were collected from all 74 participants (38 BC patients and 36 HCs) at baseline, prior to breast cancer treatment (including surgery, chemotherapy, or radiotherapy) and immediately stored at −80 °C until processing. Total genomic DNA was extracted using a commercial kit (DP712; TIANGEN Biotech Co., Ltd., Beijing, China) according to the manufacturer's protocol.

Shotgun metagenomic sequencing was performed on the Illumina Novaseq 6000 platform (Illumina Inc., San Diego, CA, USA). Raw sequencing reads were processed using the KneadData pipeline to remove low-quality bases and host-derived sequences. Quality-filtered reads were assembled into contigs using MEGAHIT with default parameters (Li et al., 2015). Metagenome-assembled genomes (MAGs) were generated by independently binning contigs using MetaBAT2 and VAMB with default settings, The resulting bin sets were subsequently integrated and dereplicated using DAS Tool, which combines outputs from multiple binning algorithms to produce optimized, non-redundant bins (Aroney et al., 2025; Kang et al., 2019; Sieber et al., 2018). The outputs from the three binning tools were integrated using a custom in-house script to maximize bin completeness and minimize redundancy.

The quality of MAGs was assessed using CheckM (Parks et al., 2015), which estimated completeness and contamination levels. Then, the MAGs were classified as high-quality (completeness ≥80%, contamination ≤ 5%), medium-quality (completeness ≥70%, contamination ≤ 10%), or partial-quality (completeness ≥50%, contamination ≤ 5%) based on these metrics (He et al., 2025). High-quality MAGs were clustered at 95% average nucleotide identity using dRep to generate species-level genome bins (SGBs), with the most complete representative genome selected from each cluster. The relative abundance of each SGB was calculated using CoverM with the following parameters: –min-read-percent-identity 0.95 and –min-cover-fraction 0.4 (Aroney et al., 2025).

### 2.3 Prediction of gut metabolic modules (GMMs), functional annotation, and bioactive metabolite profiling

Functional potential was assessed through the identification of key GMM using a previously established multi-omics framework for probiotic functional evaluation. Open reading frames were predicted from each SGB and annotated against the Kyoto Encyclopedia of Genes and Genomes database to infer metabolic pathways. The distribution of each GMM across SGBs was determined using Omixer-RPM with a correlation threshold of 0.66.

Gut bioactive metabolite profiles were predicted using the MelonnPan-predict workflow (Sun et al., 2022). For each sample, one million sequencing reads were subsampled using seqtk and aligned to reference protein databases via DIAMOND using the BLASTX algorithm (Buchfink et al., 2015). Gene abundance was calculated based on the best-hit gene alignment for each read. These abundance profiles were then transformed into predicted metabolite profiles, enabling inference of microbial metabolic activity.

### 2.4 Phageome identification and abundance quantification

The VIBRANT tool was used to identify potential phage features from contigs exceeding 1,000 bp in length (Kieft et al., 2020). The quality and completeness of predicted phage genomes were evaluated using CheckV (Nayfach et al., 2021a,b). Viral contigs longer than 5,000 bp were further processed using CD-HIT (Fu et al., 2012; available at https://github.com/weizhongli/cdhit) to cluster sequences at 95% nucleotide identity and 80% alignment coverage, resulting in the definition of viral operational taxonomic units (vOTUs).

To evaluate the novelty of viral genomes, 15,516 vOTUs were compared against 189,680 reference genomes from the Metagenomic Gut Virus catalog (accessed July 2021; Nayfach et al., 2021a,b), based on pairwise average amino acid identity (AAI) ≥90% and an alignment coverage ≥50%. The relative abundance of each vOTU was quantified using CoverM contig pipeline (https://github.com/wwood/CoverM) with the following parameters: –min-read-percent-identity 0.95, –min-read-aligned-percent 0.5, –properpairs-only, and –exclude-supplementary. Legacy morphology-based family names (e.g., *Siphoviridae, Myoviridae*, and *Microviridae*) were retained only when the corresponding phylogenetic clades have not yet been formally renamed in the ICTV database (Turner et al., 2023). This approach allows consistency with current standards while maintaining interpretability in the context of published virome literature.

### 2.5 Construction and evaluation of the BC diagnostic model

A validation cohort was constructed using publicly available fecal metagenomic data retrieved from the National Center for Biotechnology Information under BioProject accession number PRJNA453965, comprising 70 BC patients and 60 HCs. Raw sequencing data were processed using the same bioinformatics pipeline as applied to the discovery cohort, including quality control, host read removal, assembly, binning, and taxonomic profiling.

Using the microbial and metabolic features identified as differentially abundant in the discovery cohort, multiple machine learning algorithms, including Gradient Boosting Decision Tree (CatBoost), Gradient Boosting (GB), K Nearest Neighbors (KNN), Light Gradient Boosting Machine (LightGBM), Logistic Regression (LR), Multilayer Perceptron (MLP), naive Bayes (NB), Random Forest (RF), Support Vector Machine (SVM), and eXtreme Gradient Boosting (XGBoost), were trained to classify individuals as either BC patients or HCs. Model performance was evaluated using 10-fold stratified cross-validation (random state = 42) to maintain proportional class representation across folds. The area under the receiver operating characteristic curve (AUC), sensitivity, specificity, and accuracy were calculated to assess predictive performance. All machine learning analyses were implemented in Python 3.8 using scikit-learn version 0.24.2.

### 2.6 Statistical analyses

All statistical analyses and data visualization were performed using R software (version 4.3.2) and Adobe Illustrator. Demographic characteristics between groups were compared using the chi-square test. Diversity metrics were calculated based on normalized relative abundance using the R package vegan. Alpha diversity was assessed using the Shannon diversity index. Beta diversity was evaluated using Bray-Curtis dissimilarity, and principal coordinates analysis was performed to visualize intergroup differences in microbial and metabolite profiles. Partial least squares discriminant analysis was conducted using the mixOmics package to identify discriminatory features between groups. All ordination plots and data visualizations were generated using vegan and ggpubr.

Differences in microbial and metabolite abundances between groups were assessed using the Wilcoxon rank-sum test for non-normally distributed data and the *t*-test for normally distributed variables. *P*-values were adjusted for multiple testing using the Benjamini–Hochberg procedure, and a corrected *P* < 0.05 was considered statistically significant. Associations between microbial taxa and metabolites were evaluated using Spearman's rank correlation coefficient.

## 3 Results

### 3.1 Participant demographics and clinical characteristics

The demographic and clinical characteristics of the 38 BC patients and 36 HCs are summarized in Table 1. No significant differences were observed between groups in age (BC: 51.15 ± 8.78 years; HCs: 49.29 ± 9.87 years; *P* > 0.05), body mass index (BMI), educational attainment, alcohol consumption, or smoking history (*P* > 0.05). Molecular subtypes, tumor staging (I–III), and menopausal status of BC patients are detailed in Table 1.

### 3.2 Altered gut microbiota composition in BC

#### 3.2.1 Community structure and diversity

**Figure 1:**
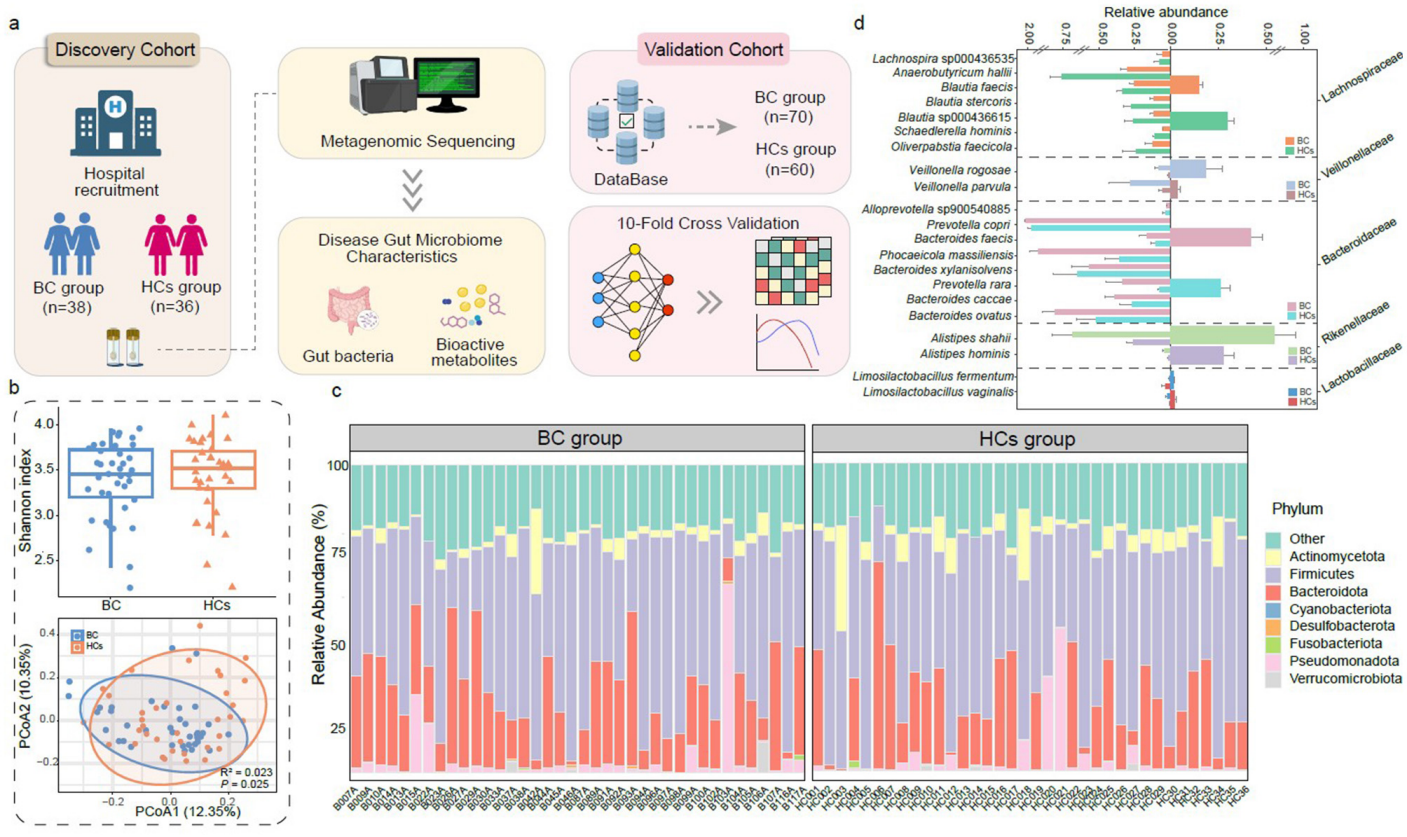
Study design and fecal metagenomic analysis of the gut microbiome in breast cancer patients and healthy controls. **(a)** Schematic workflow of participant recruitment for the discovery cohort, sample collection, metagenomic sequencing, and data analysis, with validation performed in an independent cohort. **(b)** Bacterial diversity: α diversity (Shannon index; upper panel) and β diversity (principal coordinates analysis based on Bray-Curtis dissimilarity, with Adonis test results from 999 permutations; lower panel) of gut microbiota between breast cancer (BC, *n* = 38) and healthy control (HCs, *n* = 36) groups. **(c)** Phylum-level composition: relative abundances of bacterial phyla. Unclassified species-level genome bins (SGBs) are grouped as “Other.” **(d)** Differentially abundant species: horizontal bar chart showing SBGs with significantly different abundances between groups, annotated with family-level classification (*P* < 0.05; two-sided Wilcoxon rank-sum test, Benjamini–Hochberg correction). Error bars represent standard deviation.

A schematic illustration of cohort recruitment, sample collection, and data analysis, including both the discovery cohort and the independent validation cohort is shown in Figure 1a. Metagenomic sequencing of fecal samples from 38 BC patients and 36 HCs yielded 0.50 Tbp of high-quality data (mean 6.73 Gbp per sample; Supplementary Table S1). A total of 2,470 MAGs were reconstructed, including 1,883 high-quality MAGs. Dereplication at 95% average nucleotide identity, yielded 321 SGBs. While α-diversity (Shannon index) did not differ between groups (*P* > 0.05), β-diversity analysis revealed significant microbiota structural separation (principal coordinates analysis; *R*^2^ = 0.023, *P* < 0.05; Figure 1b).

#### 3.2.2 Taxon-specific alterations

At the phylum level, eight bacterial phyla were detected across all samples, with Firmicutes (48.37%), Bacteroidota (21.60%), and Actinomycetota (4.23%) being the most abundant in both groups (Figure 1c; Supplementary Table S2). No significant differences in the relative abundances of these dominant phyla were observed (*P* > 0.05). Notably, Desulfobacterota was significantly enriched in BC patients (*P* < 0.05).

Fifty-one species exhibited significant differential abundance: 20 were enriched in BC patients, including *Prevotella copri* (family: *Prevotellaceae*), *Alistipes shahii* (*Rikenellaceae*), and *Veillonella rogosae* (*Veillonellaceae*); 31 were depleted, including *Blautia stercoris, Lachnospira* sp000436535 (both *Lachnospiraceae*), and *Limosilactobacillus fermentum* (*Lactobacillaceae*) (all *P* < 0.05; Figure 1d; Supplementary Table S3).

#### 3.2.3 Microbial diversity and composition across cancer stages

To further investigate whether gut microbial features varied across different breast cancer stages, patients were stratified into stage I (*n* = 16), stage II (*n* = 14), and stage III (*n* = 8) groups. No significant differences were observed in either α-diversity or β-diversity indices among the three stages, indicating that the overall gut microbial structure was relatively similar and stable across patients with different disease severities. Subsequent comparative analyses of microbial composition revealed specific taxa distinguishing the stages. In pairwise comparisons, 9, 19, and 65 differential SGBs were identified between stages I vs. II, II vs. III, and I vs. III, respectively. Notably, 15 SGBs (*Akkermansia muciniphila*. *Bifidobacterium pseudocatenulatum*, and, *Dialister invisus*; Supplementary Table S4) were consistented in both II vs. III, and I vs. III groups, suggesting that a stage-associated microbial signature may emerge with disease progression. While the limited sample size warrants cautious interpretation, these findings offer valuable exploratory insights that warrant confirmation in larger and more diverse cohorts.

### 3.3 Breast cancer-associated gut phageome dysbiosis

#### 3.3.1 Phageome characterization

**Figure 2:**
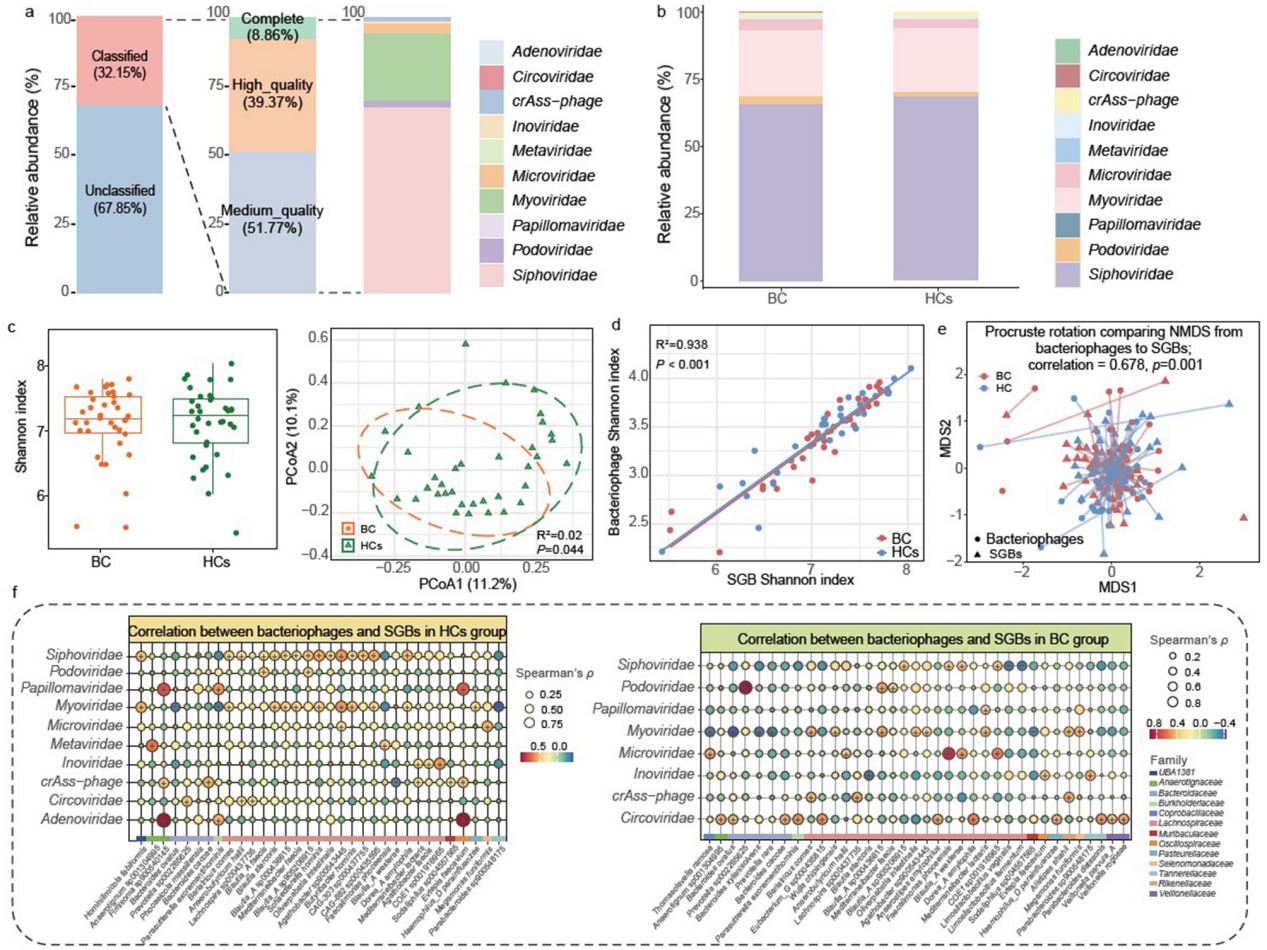
Gut phageome dysbiosis and bacteriophage-bacterial correlations in breast cancer. **(a, b)** Taxonomic and quality distribution of viral operational taxonomic units (vOTUs) in the gut phageome, annotated at the family level: **(a)** full dataset; **(b)** classified families in breast cancer (BC, *n* = 38) and healthy control (HCs, *n* = 36) groups. **(c)** Phageome diversity: α diversity (Shannon index; left panel) and β diversity (principal coordinates analysis based on Bray-Curtis dissimilarity, with Adonis test results from 999 permutations; right panel). **(d)** Pearson correlation between Shannon diversity indices of bacterial (species-level genome bin, SGB) and bacteriophage communities across all participants. **(e)** Procrustes analysis of SGB and bacteriophage community structures. **(f)** Spearman's correlation analysis between gut bacteriophage families and SGBs in the HCs and BC groups. Circle size represents the magnitude of Spearman's ρ. The color scale represents correlation direction (red: positive; blue: negative; scale shows ρ value). Symbols (+/–) within circles denote significant positive/negative correlations (*P* < 0.05); colors represent the bacterial families to which each SGB belongs.

Phageome composition plays an important role in host health. The gut phageome was characterized based on the parasitic and lytic relationships between bacteriophages and their bacterial hosts. Analysis of 15,515 non-redundant vOTUs identified 4,989 (32.16%) belonging to ten known bacteriophage families (Figure 2a). Quality assessment classified these as 442 complete (8.86%), 1,964 high-quality (39.37%), and 2,583 medium-quality (51.77%) vOTUs (Figure 2a). The top three families were *Siphoviridae* (67.04%), *Myoviridae* (24.21%), and *Microviridae* (3.63%; Figure 2b).

#### 3.3.2 Disease-associated phageome shifts

Significant β diversity differences were detected between groups (*R*^2^ = 0.02, *P* = 0.042; Figure 2c), suggesting structural divergence in phage communities and BC is associated with distinct gut virome composition, despite unchanged α-diversity (*P* > 0.05; Figure 2c). At the family level, no significant differences in phage abundance were detected. Notably, 117 vOTUs (88 *Siphoviridae*, 22 *Myoviridae*, seven *Microviridae*) showed reduced abundance in BC patients (Supplementary Table S5). Strong concordance was observed between bacterial and phage α-diversity (*R*^2^ = 0.938; *P* < 0.001; Figure 2d) and community structures (Procrustes correlation = 0.678, *P* = 0.001; Figure 2e). These results indicate a high degree of ecological co-occurrence and potential host specificity of gut phages.

### 3.4 Disrupted phage-bacteria co-occurrence networks in BC

Correlations between differentially abundant SGBs and gut phage families were analyzed in HCs and BC patients (|Spearman's ρ| > 0.3; *P* < 0.05; Figure 2f; Supplementary Table S6). In the HCs group, 54 significant correlations were identified (50 positive, four negative) involving ten phage families. In contrast, the BC group exhibited only 45 significant correlations (37 positive, eight negative) across eight phage families, indicating attenuated phage-bacteria connectivity in BC.

### 3.5 Altered gut microbial metabolic pathways and predicted metabolites in BC

#### 3.5.1 Alterations in GMMs

**Figure 3:**
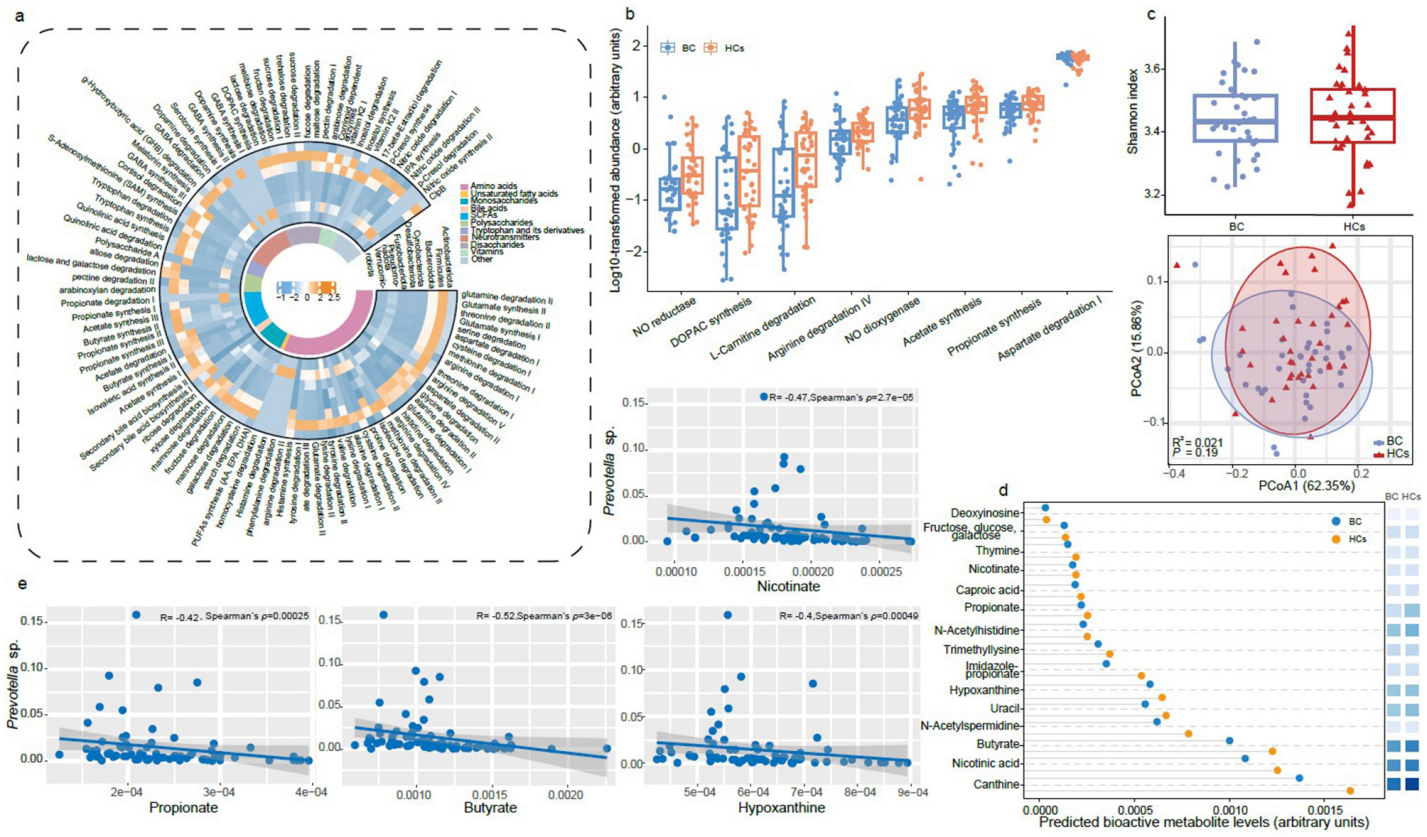
Alterations in predicted gut metabolic modules (GMMs) and bioactive metabolites in breast cancer. **(a)** Distribution of gut metabolic modules: heatmap of 98 GMMs across 11 functional categories, including amino acids, short-chain fatty acids (SCFAs), tryptophan and its derivatives, unsaturated fatty acids, carbohydrates, bile acids, neurotransmitters, vitamins, among others, encoded by eight bacterial phyla. Each category is represented by a distinct color. The color scale reflects normalized proportion of species-level genome bins encoding each module, ranging from high (orange) to low (blue) proportion. **(b)** Differentially abundant GMMs: box plots showing significantly altered metabolic modules in breast cancer (BC, *n* = 38) and healthy control (HCs, *n* = 36) groups (*P* < 0.05, two-sided Wilcoxon rank-sum test). **(c)** Predicted metabolite diversity: α diversity (Shannon index; upper panel) and β diversity (principal coordinates analysis based on Bray-Curtis dissimilarity, with Adonis test results from 999 permutations; lower panel). **(d)** Predicted bioactive metabolites significantly altered in BC patients (*P* < 0.05 in all cases, two-sided Wilcoxon rank-sum test), illustrated by the heatmap, with the color intensity represents relative abundance, ranging from high (dark blue) to low (light blue). **(e)** Correlations between *Prevotella* sp. and predicted bioactive metabolites depleted in BC: scatter plots showing negative associations (Spearman's ρ < −0.4, *P* < 0.01). The gray error band represents the fitted values, and the shaded area indicates the 95% confidence interval. NO, nitric oxide; DOPAC, dihydroxyphenylacetic acid.

Functional profiling of the 321 SGBs was performed using MetaCyc and KEGG databases (Caspi et al., 2020; Kanehisa and Goto, 2000), identifying 98 GMMs across 11 metabolic categories (Figure 3a). These modules were encoded by eight phyla, including Firmicutes (66.55%), Bacteroidota (21.17%), Pseudomonadota (5.42%), Actinobacteriota (4.75%), among others, with primary involvement in the metabolism of amino acids, SCFAs, and tryptophan and its derivatives.

Corrinoid-dependent enzymes (3.59%), acetate synthesis (3.53%), and S-adenosylmethionine (SAM) synthesis (3.48%) were identified as the three most abundant GMMs across all samples (Supplementary Table S7). Significant reductions in SCFA synthesis pathways (acetate, propionate), arginine degradation, nitric oxide degradation, and 3,4-dihydroxyphenylacetic acid (DOPAC) synthesis were observed in BC patients compared to HCs (*P* < 0.05). Conversely, aspartate degradation pathways were enriched in the BC cohort (*P* < 0.05; Figure 3b; Supplementary Table S7), suggesting a shift in nitrogen and energy metabolism associated with disease state.

#### 3.5.2 Shifts in predicted bioactive metabolites

Using the MelonnPan pipeline, 80 bioactive metabolites were predicted from metagenomic data. While no differences in α-diversity (Shannon index) or β-diversity (principal coordinates analysis) of the metabolite profile were detected between the BC and HCs groups (*P* > 0.05; Figure 3c), 15 metabolites exhibited significant abundance differences. Specifically, reductions in predicted levels of SCFAs (propionate, butyrate), nicotinate, hypoxanthine, and xanthine were prominent in BC patients (*P* < 0.05; Figure 3d; Supplementary Table S8), all of which are implicated in immune regulation, energy homeostasis, and redox balance.

Spearman correlation analysis revealed significant negative associations between *Prevotella* sp. and multiple depleted metabolites, including propionate, butyrate, xanthine, and nicotinate (Spearman's ρ < −0.4, *P* < 0.01; Figure 3e), suggesting that the overabundance of this genus in BC may contribute to an unfavorable metabolic environment. Correlation analysis further revealed multiple interconnected relationships among the microbiome, virome, and metabolites. Notably, *Siphoviridae* showed significant positive correlations with several bioactive metabolites, including nicotinate, thymine, and xanthine. In addition, bacterial taxa such as *Blautia* sp. and *Lachnospira* sp. exhibited positive associations with major SCFAs, including acetate, butyrate, and propionate, while also displaying positive correlations with *Siphoviridae* phages (Spearman's ρ > 0.5, *P* < 0.05; Supplementary Table S9).

These findings indicate that BC is associated with extensive remodeling of gut microbial metabolic pathways, characterized by depletion of immunomodulatory SCFAs and cofactor synthesis, alongside disrupted host-microbiota metabolic crosstalk.

### 3.6 Development and validation of an integrated machine learning model for BC detection using gut microbiome signatures

**Figure 4:**
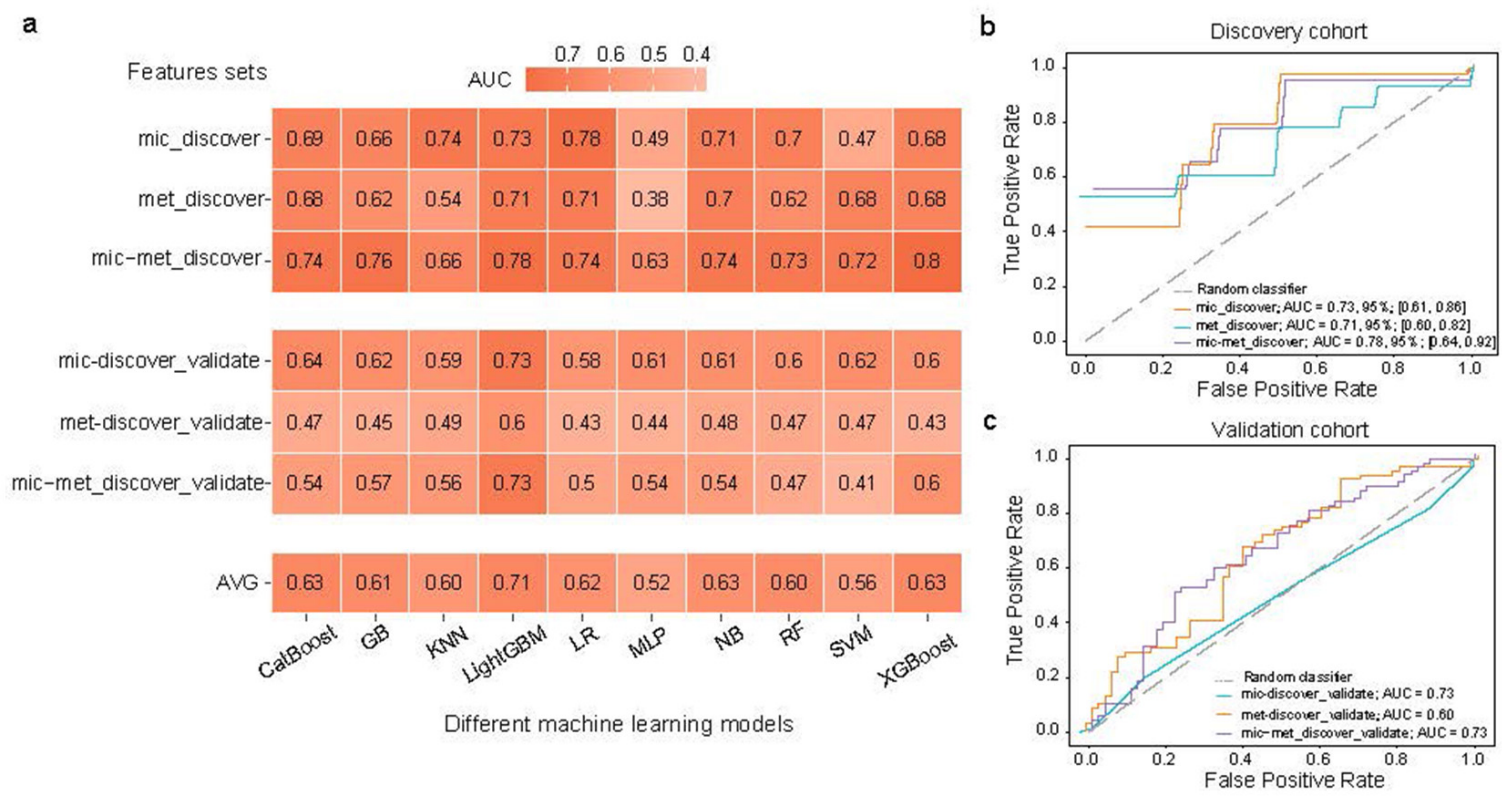
Machine learning-based diagnostic performance for breast cancer using integrated microbiome-metabolite features. **(a)** Heatmaps of area under the receiver operating characteristic curve (AUC) values for ten machine learning models using three feature sets: microbiota (mic), metabolites (met), and combined microbiota-metabolites (mic-met) in discovery cohort (_discover; *n* = 38 breast cancer patients and 36 healthy controls) and validation cohort (_validate; *n* = 70 patients and 60 controls). The color scale represents AUC magnitude (darker = higher performance). The AUC values were obtained via tenfold cross-validated. **(b, c)** Receiver operating characteristic curves illustrating model performance in the **(b)** discovery cohort and **(c)** validation cohort. Line colors denote feature sets; dashed diagonal line represents the performance of a random classifier (AUC = 0.5), indicating chance-level discrimination. CatBoost, CatBoost; GB, Gradient Boosting; KNN, K Nearest Neighbors; LightGBM, Light Gradient Boosting Machine; LR, Logistic Regression; MLP, Multilayer Perceptron; NB, naive Bayes; RF, Random Forest; SVM, Support Vector Machine; XGBoost, eXtreme Gradient Boosting.

In the discovery cohort, a predictive modeling framework was established using tenfold cross-validation to evaluate three distinct feature sets: (1) differentially abundant gut microbial species (mic_discover); (2) differentially abundant gut metabolites (met_discover); and (3) combined microbiota-metabolite features (mic-met_discover). From the pool of significantly altered microbial and metabolic features identified in the discovery cohort, a minimal 15-feature signature, comprising 11 microbial taxa and four predicted metabolites, was selected using recursive feature elimination. Feature selection and data standardization were performed within each cross-validation fold to prevent data leakage and ensure unbiased performance estimation. Model performance was assessed using the AUC, with results for both the discovery and independent validation cohorts presented in Figure 4a.

Among the ten machine learning algorithms evaluated, LightGBM initially demonstrated the highest baseline performance and was selected for in-depth analysis. Diagnostic performance of the three feature sets was assessed using tenfold cross-validation in the discovery cohort. Surprisingly, LightGBM classifier outperformed the other models when applied to a model that included the microbiota-only, metabolites-only, and both (Average AUC = 0.71; Figure 4a), leading us to select LightGBM for subsequent analysis. The AUC of LightGBM was 0.73 (95% CI: [0.61, 0.86]) when applied to the microbiota-only model. A comparable performance was observed for the metabolite-only model, which yielded an AUC of 0.71 (95% CI: [0.60, 0.82]). Notably, the integration of microbial and predicted metabolite features substantially enhanced diagnostic accuracy, resulting in an AUC of 0.78 (95% CI: [0.64, 0.92]; Figure 4b). These results indicate that combining taxonomic and functional profiles improves the model's ability to discriminate between BC patients and HCs.

Generalizability was subsequently assessed in an independent validation cohort (*n* = 130; Figure 4c): mic_discover_validate (microbial features), met_ours_validate (metabolite features), and mic-met_discover_validate (combined features). Robust performance was maintained for the integrated signature (mic-met*_*discover_validate), with an AUC of 0.73, equivalent to microbiota-only features (AUC of 0.73) and superior to metabolite-only features (AUC of 0.60). This combined model demonstrated optimal recall (0.74) and accuracy (0.69) metrics. The preserved diagnostic capability across cohorts confirms the utility of integrated metagenomic-metabolomic signatures as non-invasive biomarker for auxiliary BC detection and risk stratification.

## 4 Discussion

Breast cancer remains the most frequently diagnosed malignancy and a leading cause of cancer-related mortality among women worldwide. While genetic and environmental factors are established contributors, they are insufficient to fully account for disease risk. In recent years, the gut microbiota has emerged as a critical modulator of host physiology, with dual roles in maintaining health and influencing disease pathogenesis, effects that are increasingly attributed to shifts in microbial composition and function. Given its involvement in immune regulation, metabolic signaling, and inflammatory control, the gut microbiome has been implicated as a potential contributor to BC development and progression (Zeng et al., 2019; Zhu et al., 2018).

In this study, although no significant differences in α-diversity were observed between BC patients and HCs, distinct alterations in gut microbial composition were identified, indicating that taxonomic shifts, not overall diversity, may be more relevant to disease state. Several beneficial bacterial taxa were significantly depleted in BC patients, including *L. fermentum, Lachnospira* sp., *Blautia* sp., *Parabacteroides* sp., and *Prevotella* sp. *Limosilactobacillus fermentum*, a well-characterized lactic acid bacterium, has been proposed as a next-generation probiotic due to its ability to produce exopolysaccharides and bacteriocins, modulate gut immunity, and stimulate immunoglobulin secretion, thereby attenuating mucosal inflammation (Naghmouchi et al., 2020). Preclinical evidence has demonstrated that *L. fermentum* enhances lymphocyte proliferation and activates immune effector cells, counteracting immunosuppression in cyclophosphamide-treated murine models (Kim et al., 2023). The depletion of *Blautia* sp. in BC patients is particularly noteworthy, as a prior study has linked reduced abundance of this genus to advanced clinical stage and histoprognostic grade in BC suggesting a potential protective role (Luu et al., 2017).

Conversely, taxa associated with pro-inflammatory or pathogenic potential were enriched in the BC group, including *Megamonas funiformis, Veillonella parvula*, and *Parasutterella excrementihominis, Megamonas funiformis* and *Veillonella parvula* are opportunistic pathogens previously reported to be elevated in individuals with inflammatory bowel disease and colorectal cancer (Ren et al., 2020; Sakon et al., 2008). *Veillonella parvula*, commonly found in the oral cavity, respiratory tract, and gut, has been associated with advanced tumor stage, lymph node metastasis, and poor prognosis in colorectal cancer, potentially promoting carcinogenesis through activation of immune pathways involving B lymphocyte stimulator expression and B-cell infiltration (Qian et al., 2023). Furthermore, *Prevotella copri*, the most prevalent species within the *Prevotella* genus in the human gut, was significantly enriched in BC patients, consistent with findings by Su et al. (2024), who demonstrated that oral administration of *P. copri* promoted tumor growth in specific pathogen-free mice. This effect was attributed to excessive tryptophan consumption by *P. copri*, leading to reduced levels of indole-3-pyruvic acid, a metabolite with anti-inflammatory and antioxidant properties (Roager and Licht, 2018), thereby creating a microenvironment conducive to tumor progression. In the present study, *Prevotella* sp. were also found to be negatively correlated with beneficial microbial metabolites such as acetate, butyrate, and nicotinate (vitamin B3), suggesting that their overabundance may indirectly facilitate carcinogenesis through metabolic disruption.

In our stratified analysis of breast cancer patients across clinical stages I–III, no significant differences were observed in overall gut microbial diversity. Nevertheless, pairwise comparisons revealed several stage-associated taxa, including *A. muciniphila, B. pseudocatenulatum*, and *Alistipes shahii*, which were depleted in patients with advanced disease. These microbes have been implicated in mucin degradation, immune modulation, and lipid metabolism. Although these findings point to a potential stage-related microbial shift, the small sample size within each subgroup limits statistical robustness. Larger, longitudinal cohorts are needed to confirm these preliminary associations.

The gut virome, though less characterized than the bacterial microbiome, plays a crucial role in shaping microbial community structure through bacteriophage-mediated lysis and horizontal gene transfer (Ogilvie and Jones, 2015). Despite advances in metagenomic assembly, a substantial proportion of gut phages remain unclassified and are often referred to as “viral dark matter” (Stockdale and Hill, 2021). In healthy adults, the dominant bacteriophage families typically comprise *Siphoviridae, Myoviridae, Podoviridae*, and *Microviridae*. Notably, *Siphoviridae* has been proposed as a potential viral biomarker in neurodegenerative conditions such as Alzheimer's disease and has been associated with cognitive performance (Ghorbani et al., 2023; Zhao et al., 2025). Concurrent with bacterial dysbiosis, significant restructuring of the gut phageome was identified between BC patients and HCs in this study, despite the absence of significant differences in α diversity. Dominant phage families (*Siphoviridae, Myoviridae, Microviridae*) exhibited altered abundances, with 117 vOTUs significantly reduced in BC patients. Furthermore, strong correlations between bacterial and phage α-diversity (*R*^2^ = 0.938) and community structures (Procrustes correlation = 0.678, *P* = 0.001) suggest intricate phage-bacteria interactions and coordinated ecological dynamics. Furthermore, we observed significant positive correlations between *Siphoviridae* phages and several bioactive metabolites, including nicotinate, thymine, and xanthine. This suggests that the gut virome may indirectly regulate the host metabolic by modulating the abundance or functional activity of specific bacterial taxa. Consistently, recent studies have shown that phages can encode auxiliary metabolic genes, influence lipid and nucleotide biosynthetic pathways, and drive shifts in microbial metabolic associated with cancer progression (Benler et al., 2021; Hannigan et al., 2018). These findings indicate that BC is associated with disruption of phage-bacteria interactions, potentially contributing to microbial dysbiosis and potential destabilization of microbial ecology in cancer states; however, the functional implications of these virome alterations remain to be fully elucidated.

These taxonomic shifts were associated with significant functional metabolic perturbations. A marked reduction was observed in the predicted abundance of SCFAs, particularly propionate and butyrate. Butyrate is known to exert anti-tumor effects through multiple mechanisms, including activation of the GPR109A receptor to promote regulatory T cell differentiation, suppression of proinflammatory cytokines, and epigenetic regulation of tumor suppressor and anti-inflammatory genes via histone deacetylase inhibition (Cuadrado et al., 2018; Li et al., 2025). Preclinical research has shown that dietary butyrate supplementation suppresses tumor growth in murine models of diet-induced BC (Belobrajdic and McIntosh, 2000), and low gut SCFA levels in premenopausal obese women have been linked to increased BC risk (Ellulu et al., 2016; Rosenberg et al., 2006). In parallel, microbial pathways involved in acetate and propionate synthesis were significantly downregulated in BC patients, further supporting impaired SCFA production in the disease state. Notably, our multi-omics integration revealed that *Blautia* sp. and *Lachnospira* sp., both known SCFA-producing taxa, were positively correlated with increased levels of acetate, butyrate, and propionate. These bacteria also exhibited positive associations with Siphoviridae phages, suggesting that the gut virome may influence the metabolic capacity of key SCFAs producers by modulating their abundance or activity. Bacteriophages can reshape bacterial community structure and thereby alter host metabolic profiles. For example, phage-mediated predation on *C. sporogenes* has been shown to reduce tryptamine production, a microbiota-derived metabolite known to accelerate intestinal motility (Bhattarai et al., 2018; Hsu et al., 2019). Additionally, perturbations in purine and nucleotide metabolism were evident, with reduced levels of key intermediates in the purine salvage pathway (hypoxanthine and xanthine) as well as nucleic acid precursors such as uracil and thymine. Given the high nucleotide demand of rapidly proliferating cancer cells, efficient recycling via the salvage pathway may confer a metabolic advantage (Jovicic, 2024). Tran et al. (2024) found that cancer cells exploit both *de novo* synthesis and salvage pathways to sustain nucleotide pools, and depletion of salvage intermediates has been recognized as a metabolic hallmark in cancers including non-small cell lung cancer and pancreatic ductal adenocarcinoma. The observed reduction in these metabolites likely reflects their preferential consumption by tumor cells, representing a signature of metabolic reprogramming and highlighting their potential as non-invasive biomarkers in BC (Battelli et al., 2016; Kang et al., 2024).

Altered amino acid metabolism is recognized as a critical metabolic adaptation supporting cancer cell proliferation. Significant amino acid perturbations in the urine, serum, and breast tissue have been consistently reported in BC patients (Bel'skaya et al., 2021; Wu et al., 2024), suggesting systemic metabolic reprogramming associated with disease progression. Our findings further substantiate this, as a reduction in the gut microbial arginine degradation module and an increase in aspartate degradation were observed in BC patients. Elevated arginine demand is a known hallmark of tumor metabolism, consistent with reports of increased arginase-1, an enzyme that catalyzes arginine catabolism, in both the serum and saliva of BC patients, which is associated with immunosuppression and adverse clinical outcomes (Perez et al., 2012; Polat et al., 2003). Arginine also serves as a primary substrate for nitric oxide synthesis, a signaling molecule implicated in tumor angiogenesis, metastasis, resistance to apoptosis, and immune evasion. Concordantly, increased nitric oxide synthase expression and nitric oxide levels, linked to vascular endothelial growth factor-driven angiogenesis and metastasis, have been reported in cancer patients (Bronte and Zanovello, 2005; Chen et al., 2021; Mocellin et al., 2007). This aligns with the reduction in nitric oxide degradation modules (I and II) observed in our BC cohort, potentially contributing to a pro-tumorigenic environment through the accumulation of bioactive nitric oxide. These results suggest that gut microbial metabolism may indirectly influence host nitrogen metabolism and redox signaling in ways that favor tumor progression.

The clinical imperative for non-invasive breast cancer diagnostics is addressed by our development of a machine learning model integrating species-level microbiota features and predicted bioactive metabolites. This multi-omics approach demonstrated superior diagnostic performance (AUC = 0.80) compared to models utilizing microbiota or metabolite features alone. Robust generalizability was confirmed in an independent validation cohort (AUC = 0.73), with consistent performance across demographic subgroups, positioning it as a promising strategy for non-invasive BC risk stratification. Although viral signatures were evaluated, they did not improve model performance beyond bacterial and metabolite features (Data not shown); thus, they were not included in the final model. We acknowledge that the current phageome characterization relied on metagenomic sequencing and reference-based annotation, which poses challenges due to the extensive genomic diversity of bacteriophages and the ongoing refinement of viral taxonomies. These methodological constraints may lead to reduced detection sensitivity and underestimation of the virome's contribution to host physiology. Future studies employing virome-enriched sequencing and larger cohorts may further elucidate the contribution of viral features to disease prediction. The enhanced predictive capability underscores the clinical value of leveraging microbiome-host metabolic crosstalk for early cancer detection.

Several limitations of our study must be acknowledged. Breast cancer is a heterogeneous disease influenced by intricate hormonal interactions and diverse molecular subtypes, presents analytical challenges. The relatively small sample size precluded meaningful subgroup analyses stratified by critical clinical parameters such as hormone receptor status, molecular classification, or disease stage. Although baseline characteristics were well-matched between groups, the observed attenuation in predictive accuracy within the validation cohort likely reflects inherent inter-sample and inter-cohort heterogeneity. Therefore, future studies with larger, multi-center cohorts are warranted to validate these findings and improve the generalizability of the predictive model. Viral taxonomic annotation remains challenging due to ongoing updates in ICTV classification, and legacy morphology-based names were retained only when formal phylogenetic reassignment was not yet available, which may limit the precision of virome interpretation. Furthermore, while bioactive metabolites and functional profiles were predicted computationally, these findings remain inferential. Experimental validation through targeted metabolomics, metaproteomics, and *in vitro* or *in vivo* functional assays will be essential to confirm the biological relevance of identified metabolites and establish their mechanistic links to disease pathogenesis.

In conclusion, characteristic alterations in gut microbial composition and predicted bioactive metabolite profiles were identified through metagenomic sequencing of fecal samples from BC patients and HCs. These features were leveraged to construct a machine learning diagnostic model, wherein the integration of microbiota and metabolite data was found to effectively distinguish BC patients from HCs, with strong performance in both discovery and validation settings. This work provides a foundational basis for developing non-invasive, microbiome-based strategies for the early diagnosis of breast cancer. This study comprehensively characterizes distinct alterations in the gut microbiome, phageome, and associated metabolic landscape in BC patients compared to HCs. We identified significant taxonomic shifts, including depletion of potentially beneficial bacteria (e.g., *L. fermentum, Blautia* sp.) and enrichment of taxa linked to pathogenesis (e.g., *Prevotella copri, Veillonella parvula*). Concurrently, the gut phageome exhibited reduced diversity and disrupted correlations with bacterial hosts. Functional metagenomic profiling revealed critical metabolic perturbations, notably reduced synthesis pathways for immunomodulatory SCFAs (acetate, propionate, butyrate) and depletion of key metabolites like butyrate, hypoxanthine, and xanthine. Crucially, we translated these findings into a clinically applicable tool. By integrating species-level microbial features and predicted bioactive metabolites, a machine learning model demonstrated high diagnostic accuracy (AUC = 0.78) in distinguishing BC patients from HCs. The robustness of this multi-omics signature was confirmed in an independent validation cohort (AUC = 0.73). These results establish the gut microbiome-metabolite axis as a powerful source of non-invasive biomarkers. This work provides a foundational framework for developing gut microbiota-based diagnostic strategies to improve the early detection of BC, warranting further validation in larger, molecularly stratified cohorts and functional mechanistic studies.

## Data Availability

The datasets presented in this study can be found in online repositories. The names of the repository/repositories and accession number(s) can be found below: https://db.cngb.org/data_resources/?query=CNP0007592, CNP0007592.

## References

[B1] Aroney S. T. N. Newell R. J. P. Nissen J. N. Camargo A. P. Tyson G. W. Woodcroft B. J. . (2025). CoverM: read alignment statistics for metagenomics. Bioinformatics 41:btaf147. doi: 10.1093/bioinformatics/btaf147 40193404 PMC11993303

[B2] Battelli M. G. Polito L. Bortolotti M. Bolognesi A. (2016). Xanthine oxidoreductase in cancer: more than a differentiation marker. Cancer Med. 5, 546–557. doi: 10.1002/cam4.601 26687331 PMC4799950

[B3] Belobrajdic D. P. McIntosh G. H. (2000). Dietary butyrate inhibits NMU-induced mammary cancer in rats. Nutr. Cancer 36, 217–223. doi: 10.1207/S15327914NC3602_11 10890033

[B4] Bel'skaya L. V. Sarf E. A. Kosenok V. K. (2021). Indicators of L-arginine metabolism in saliva: a focus on breast cancer. J. Oral Biosci. 63, 52–57. doi: 10.1016/j.job.2020.12.002 33476704

[B5] Benler S. Yutin N. Antipov D. Rayko M. Shmakov S. Gussow A. B. . (2021). Thousands of previously unknown phages discovered in whole-community human gut metagenomes. Microbiome 9:78. doi: 10.1186/s40168-021-01017-w 33781338 PMC8008677

[B6] Bhattarai Y. Williams B. B. Battaglioli E. J. Whitaker W. R. Till L. Grover M. . (2018). Gut microbiota-produced tryptamine activates an epithelial G-protein-coupled receptor to increase colonic secretion. Cell Host Microbe 23, 775–785.e5. doi: 10.1016/j.chom.2018.05.004 29902441 PMC6055526

[B7] Bray F. Laversanne M. Sung H. Ferlay J. Siegel R. L. Soerjomataram I. . (2024). Global cancer statistics 2022: GLOBOCAN estimates of incidence and mortality worldwide for 36 cancers in 185 countries. CA Cancer J. Clin. 74, 229–263. doi: 10.3322/caac.21834 38572751

[B8] Bronte V. Zanovello P. (2005). Regulation of immune responses by L-arginine metabolism. Nat. Rev. Immunol. 5, 641–654. doi: 10.1038/nri1668 16056256

[B9] Buchfink B. Xie C. Huson D. H. (2015). Fast and sensitive protein alignment using DIAMOND. Nat. Methods 12, 59–60. doi: 10.1038/nmeth.3176 25402007

[B10] Caspi R. Billington R. Keseler I. M. Kothari A. Krummenacker M. Midford P. E. . (2020). The MetaCyc database of metabolic pathways and enzymes - a 2019 update. Nucleic Acids Res. 48, D445–D453. doi: 10.1093/nar/gkz862 31586394 PMC6943030

[B11] Chen C.-L. Hsu S.-C. Ann D. K. Yen Y. Kung H.-J. (2021). Arginine signaling and cancer metabolism. Cancers 13:3541. doi: 10.3390/cancers13143541 34298755 PMC8306961

[B12] Crusz S. M. Balkwill F. R. (2015). Inflammation and cancer: advances and new agents. Nat. Rev. Clin. Oncol. 12, 584–596. doi: 10.1038/nrclinonc.2015.105 26122183

[B13] Cuadrado A. Manda G. Hassan A. Alcaraz M. J. Barbas C. Daiber A. . (2018). Transcription factor NRF2 as a therapeutic target for chronic diseases: a systems medicine approach. Pharmacol. Rev. 70, 348–383. doi: 10.1124/pr.117.014753 29507103

[B14] D'Amico F. Perrone A. M. Rampelli S. Coluccelli S. Barone M. Ravegnini G. . (2021). Gut microbiota dynamics during chemotherapy in epithelial ovarian cancer patients are related to therapeutic outcome. Cancers 13:3999. doi: 10.3390/cancers13163999 34439153 PMC8393652

[B15] Edwinson A. L. Yang L. Peters S. Hanning N. Jeraldo P. Jagtap P. . (2022). Gut microbial β-glucuronidases regulate host luminal proteases and are depleted in irritable bowel syndrome. Nat. Microbiol. 7, 680–694. doi: 10.1038/s41564-022-01103-1 35484230 PMC9081267

[B16] Ellulu M. S. Khaza'ai H. Rahmat A. Patimah I. Abed Y. (2016). Obesity can predict and promote systemic inflammation in healthy adults. Int. J. Cardiol. 215, 318–324. doi: 10.1016/j.ijcard.2016.04.089 27128554

[B17] Fassarella M. Blaak E. E. Penders J. Nauta A. Smidt H. Zoetendal E. G. . (2021). Gut microbiome stability and resilience: elucidating the response to perturbations in order to modulate gut health. Gut 70, 595–605. doi: 10.1136/gutjnl-2020-321747 33051190

[B18] Fu L. Niu B. Zhu Z. Wu S. Li W. J. B. (2012). CD-HIT: accelerated for clustering the next-generation sequencing data. Bioinformatics 28, 3150–3152. doi: 10.1093/bioinformatics/bts565 23060610 PMC3516142

[B19] Funk J. L. Wertheim B. C. Frye J. B. Blew R. M. Nicholas J. S. Chen Z. . (2023). Association of ß-glucuronidase activity with menopausal status, ethnicity, adiposity, and inflammation in women. Menopause 30, 186–192. doi: 10.1097/GME.0000000000002106 36696643 PMC9886315

[B20] Ghorbani M. Ferreira D. Maioli S. (2023). A metagenomic study of gut viral markers in amyloid-positive Alzheimer's disease patients. Alzheimers Res. Ther. 15:141. doi: 10.1186/s13195-023-01285-8 37608325 PMC10464408

[B21] Goedert J. J. Hua X. Bielecka A. Okayasu I. Milne G. L. Jones G. S. . (2018). Postmenopausal breast cancer and oestrogen associations with the IgA-coated and IgA-noncoated faecal microbiota. Br. J. Cancer 118, 471–479. doi: 10.1038/bjc.2017.435 29360814 PMC5830593

[B22] Hannigan G. D. Duhaime M. B. Ruffin M. T. T. Koumpouras C. C. Schloss P. D. (2018). Diagnostic potential and interactive dynamics of the colorectal cancer virome. mBio 9:e02248-18. doi: 10.1128/mBio.02248-18 30459201 PMC6247079

[B23] He Q. Sun Y. Jin H. Kwok L.-Y. Ma T. Quan K. . (2025). Deciphering gut microbial dynamics and multi-omics changes in yogurt-based dietary interventions. Sci. Bull. S2095-9273(25)00535-3. doi: 10.1016/j.scib.2025.05.021 40518329

[B24] Hsu B. B. Gibson T. E. Yeliseyev V. Liu Q. Lyon L. Bry L. . (2019). Dynamic modulation of the gut microbiota and metabolome by bacteriophages in a mouse model. Cell Host Microbe 25, 803–814.e5. doi: 10.1016/j.chom.2019.05.001 31175044 PMC6579560

[B25] Jin H. Quan K. You L. Kwok L.-Y. Ma T. Li Y. . (2025). A genomic compendium of cultivated food-derived lactic acid bacteria unveils their contributions to human health. Sci. Bull. 70, 1761–1765. doi: 10.1016/j.scib.2024.12.002 39672713

[B26] Jovicic S. M. (2024). Uncovering novel therapeutic targets in glucose, nucleotides and lipids metabolism during cancer and neurological diseases. Int. J. Immunopathol. Pharmacol. 38:3946320241250293. doi: 10.1177/03946320241250293 38712748 PMC11080811

[B27] Kanehisa M. Goto S. (2000). KEGG: kyoto encyclopedia of genes and genomes. Nucleic Acids Res. 28, 27–30. doi: 10.1093/nar/28.1.27 10592173 PMC102409

[B28] Kang D. D. Li F. Kirton E. Thomas A. Egan R. An H. . (2019). MetaBAT 2: an adaptive binning algorithm for robust and efficient genome reconstruction from metagenome assemblies. PeerJ 7:e7359. doi: 10.7717/peerj.7359 31388474 PMC6662567

[B29] Kang M. Lee B. Han H.-S. He J. Kim C. H. Yoon Y.-S. . (2024). Feasibility of early diagnosis of pancreatic cancer using reagent for analyzing purine metabolite (Hypoxanthine, Xanthine) in urine. Research Square [Preprint]. doi: 10.21203/rs.3.rs-5011761/v1

[B30] Kieft K. Zhou Z. Anantharaman K. (2020). VIBRANT: automated recovery, annotation and curation of microbial viruses, and evaluation of viral community function from genomic sequences. Microbiome 8:90. doi: 10.1186/s40168-020-00867-0 32522236 PMC7288430

[B31] Kim J. Harper A. McCormack V. Sung H. Houssami N. Morgan E. . (2025). Global patterns and trends in breast cancer incidence and mortality across 185 countries. Nat. Med. 31, 1154–1162. doi: 10.1038/s41591-025-03502-3 39994475

[B32] Kim S. Lee H. H. Kang C.-H. Kang H. Cho H. (2023). Immune-enhancing effects of *Limosilactobacillus fermentum* in BALB/c mice immunosuppressed by cyclophosphamide. Nutrients 15:1038. doi: 10.3390/nu15041038 36839396 PMC9961842

[B33] Li D. Liu C.-M. Luo R. Sadakane K. Lam T.-W. J. B. (2015). MEGAHIT: an ultra-fast single-node solution for large and complex metagenomics assembly via succinct de Bruijn graph. Bioinformatics 31, 1674–1676. doi: 10.1093/bioinformatics/btv033 25609793

[B34] Li S. Wang L. Han M. Fan H. Tang H. Gao H. . (2025). Combination of sodium butyrate and immunotherapy in glioma: regulation of immunologically hot and cold tumors via gut microbiota and metabolites. Front. Immunol. 16:1532528. doi: 10.3389/fimmu.2025.1532528 40297576 PMC12035444

[B35] Luu T. H. Michel C. Bard J.-M. Dravet F. Nazih H. Bobin-Dubigeon C. . (2017). Intestinal proportion of *Blautia* sp. is associated with clinical stage and histoprognostic grade in patients with early-stage breast cancer. Nutr. Cancer 69, 267–275. doi: 10.1080/01635581.2017.1263750 28094541

[B36] Ma W. Zhang L. Chen W. Chang Z. Tu J. Qin Y. . (2024). Microbiota enterotoxigenic *Bacteroides fragilis*-secreted BFT-1 promotes breast cancer cell stemness and chemoresistance through its functional receptor NOD1. Protein Cell 15, 419–440. doi: 10.1093/procel/pwae005 38437016 PMC11131025

[B37] Mocellin S. Bronte V. Nitti D. (2007). Nitric oxide, a double edged sword in cancer biology: searching for therapeutic opportunities. Med. Res. Rev. 27, 317–352. doi: 10.1002/med.20092 16991100

[B38] Naghmouchi K. Belguesmia Y. Bendali F. Spano G. Seal B. S. Drider D. . (2020). *Lactobacillus fermentum*: a bacterial species with potential for food preservation and biomedical applications. Crit. Rev. Food Sci. Nutr. 60, 3387–3399. doi: 10.1080/10408398.2019.1688250 31729242

[B39] Nayfach S. Camargo A. P. Schulz F. Eloe-Fadrosh E. Roux S. Kyrpides N. C. . (2021a). CheckV assesses the quality and completeness of metagenome-assembled viral genomes. Nat. Biotechnol. 39, 578–585. doi: 10.1038/s41587-020-00774-7 33349699 PMC8116208

[B40] Nayfach S. Páez-Espino D. Call L. Low S. J. Sberro H. Ivanova N. N. . (2021b). Metagenomic compendium of 189,680 DNA viruses from the human gut microbiome. Nat. Microbiol. 6, 960–970. doi: 10.1038/s41564-021-00928-6 34168315 PMC8241571

[B41] Ogilvie L. A. Jones B. V. (2015). The human gut virome: a multifaceted majority. Front. Microbiol. 6:918. doi: 10.3389/fmicb.2015.00918 26441861 PMC4566309

[B42] Parks D. H. Imelfort M. Skennerton C. T. Hugenholtz P. Tyson G. W. J. G. R. (2015). CheckM: assessing the quality of microbial genomes recovered from isolates, single cells, and metagenomes. Genome Res. 25, 1043–1055. doi: 10.1101/gr.186072.114 25977477 PMC4484387

[B43] Perez G. Olivares I. M. Rodriguez M. G. Ceballos G. M. Garcia Sanchez J. R. (2012). Arginase activity in patients with breast cancer: an analysis of plasma, tumors, and its relationship with the presence of the estrogen receptor. Oncol. Res. Treat. 35, 570–574. doi: 10.1159/000343005 23038227

[B44] Polat M. F. Taysi S. Polat S. Böyük A. Bakan E. (2003). Elevated serum arginase activity levels in patients with breast cancer. Surg. Today 33, 655–661. doi: 10.1007/s00595-002-2563-2 12928840

[B45] Qian Y. Kang Z. Zhao L. Chen H. Zhou C. Gao Q. . (2023). Berberine might block colorectal carcinogenesis by inhibiting the regulation of B-cell function by *Veillonella parvula*. Chin. Med. J. 136, 2722–2731. doi: 10.1097/CM9.0000000000002752 37553874 PMC10684188

[B46] Ren X. Xu J. Zhang Y. Chen G. Zhang Y. Huang Q. . (2020). Bacterial alterations in post-cholecystectomy patients are associated with colorectal cancer. Front. Oncol. 10:1418. doi: 10.3389/fonc.2020.01418 32903396 PMC7434860

[B47] Roager H. M. Licht T. R. (2018). Microbial tryptophan catabolites in health and disease. Nat. Commun. 9:3294. doi: 10.1038/s41467-018-05470-4 30120222 PMC6098093

[B48] Rosenberg L. U. Einarsdóttir K. Friman E. I. Wedrén S. Dickman P. W. Hall P. . (2006). Risk factors for hormone receptor-defined breast cancer in postmenopausal women. Cancer Epidemiol. Biomarkers Prev. 15, 2482–2488. doi: 10.1158/1055-9965.EPI-06-0489 17164374

[B49] Sakon H. Nagai F. Morotomi M. Tanaka R. (2008). *Sutterella parvirubra* sp. nov. and *Megamonas funiformis* sp. nov., isolated from human faeces. Int. J. Syst. Evol. Microbiol. 58, 970–975. doi: 10.1099/ijs.0.65456-0 18398204

[B50] Sári Z. Mikó E. Kovács T. Jankó L. Csonka T. Lente G. . (2020). Indolepropionic acid, a metabolite of the microbiome, has cytostatic properties in breast cancer by activating AHR and PXR receptors and inducing oxidative stress. Cancers 12:2411. doi: 10.3390/cancers12092411 32854297 PMC7565149

[B51] Sieber C. M. K. Probst A. J. Sharrar A. Thomas B. C. Hess M. Tringe S. G. . (2018). Recovery of genomes from metagenomes via a dereplication, aggregation and scoring strategy. Nat. Microbiol. 3, 836–843. doi: 10.1038/s41564-018-0171-1 29807988 PMC6786971

[B52] Stockdale S. R. Hill C. (2021). Progress and prospects of the healthy human gut virome. Curr. Opin. Virol. 51, 164–171. doi: 10.1016/j.coviro.2021.10.001 34742036

[B53] Su J. Lin X. Li D. Yang C. Lv S. Chen X. . (2024). Prevotella copri exhausts intrinsic indole-3-pyruvic acid in the host to promote breast cancer progression: inactivation of AMPK via UHRF1-mediated negative regulation. Gut Microbes 16:2347757. doi: 10.1080/19490976.2024.2347757 38773738 PMC11123460

[B54] Sun B. Ma T. Li Y. Yang N. Li B. Zhou X. . (2022). Bifidobacterium lactis Probio-M8 adjuvant treatment confers added benefits to patients with coronary artery disease via target modulation of the gut-heart/-brain axes. mSystems 7:e0010022. doi: 10.1128/msystems.00100-22 35343796 PMC9040731

[B55] Sun Y. S. Zhao Z. Yang Z. N. Xu F. Lu H. J. Zhu Z. Y. . (2017). Risk factors and preventions of breast cancer. Int. J. Biol. Sci. 13, 1387–1397. doi: 10.7150/ijbs.21635 29209143 PMC5715522

[B56] Terrisse S. Derosa L. Iebba V. Ghiringhelli F. Vaz-Luis I. Kroemer G. . (2021). Intestinal microbiota influences clinical outcome and side effects of early breast cancer treatment. Cell Death Differ. 28, 2778–2796. doi: 10.1038/s41418-021-00784-1 33963313 PMC8408230

[B57] Tran D. H. Kim D. Kesavan R. Brown H. Dey T. Soflaee M. H. . (2024). *De novo* and salvage purine synthesis pathways across tissues and tumors. Cell 187, 3602–3618.e20. doi: 10.1016/j.cell.2024.05.011 38823389 PMC11246224

[B58] Turner D. Shkoporov A. N. Lood C. Millard A. D. Dutilh B. E. Alfenas-Zerbini P. . (2023). Abolishment of morphology-based taxa and change to binomial species names: 2022 taxonomy update of the ICTV bacterial viruses subcommittee. Arch. Virol. 168:74. doi: 10.1007/s00705-022-05694-2 36683075 PMC9868039

[B59] Wang H. Rong X. Zhao G. Zhou Y. Xiao Y. Ma D. . (2022). The microbial metabolite trimethylamine N-oxide promotes antitumor immunity in triple-negative breast cancer. Cell Metab. 34, 581–594.e8. doi: 10.1016/j.cmet.2022.02.010 35278352

[B60] Woelfel S. Silva M. S. Stecher B. (2024). Intestinal colonization resistance in the context of environmental, host, and microbial determinants. Cell Host Microbe 32, 820–836. doi: 10.1016/j.chom.2024.05.002 38870899

[B61] Wu H. C. Lai Y. Liao Y. Deyssenroth M. Miller G. W. Santella R. M. . (2024). Plasma metabolomics profiles and breast cancer risk. Breast Cancer Res. 26:141. doi: 10.1186/s13058-024-01896-5 39385226 PMC11463119

[B62] Yu L. C.-H. Wang J.-T. Wei S.-C. Ni Y.-H. (2012). Host-microbial interactions and regulation of intestinal epithelial barrier function: from physiology to pathology. World J. Gastrointest. Pathophysiol. 3:27. doi: 10.4291/wjgp.v3.i1.27 22368784 PMC3284523

[B63] Zeng H. Umar S. Rust B. Lazarova D. Bordonaro M. (2019). Secondary bile acids and short chain fatty acids in the colon: a focus on colonic microbiome, cell proliferation, inflammation, and cancer. Int. J. Mol. Sci. 20:1214. doi: 10.3390/ijms20051214 30862015 PMC6429521

[B64] Zhao Y. Xiong C. Wang B. Li D. Liu J. Wei S. . (2025). The discovery of phages in the substantia nigra and its implication for Parkinson's disease. Research 8:657. doi: 10.34133/research.0657 40308709 PMC12041648

[B65] Zhu B. Wang X. Li L. (2010). Human gut microbiome: the second genome of human body. Protein Cell 1, 718–725. doi: 10.1007/s13238-010-0093-z 21203913 PMC4875195

[B66] Zhu J. Liao M. Yao Z. Liang W. Li Q. Liu J. . (2018). Breast cancer in postmenopausal women is associated with an altered gut metagenome. Microbiome 6:136. doi: 10.1186/s40168-018-0515-3 30081953 PMC6080540

